# White matter in prolonged glucocorticoid response to psychological stress in schizophrenia

**DOI:** 10.1038/s41386-021-01077-4

**Published:** 2021-07-01

**Authors:** Eric L. Goldwaser, Joshua Chiappelli, Mark D. Kvarta, Xiaoming Du, Zachary B. Millman, Bhim M. Adhikari, Hugh O’Neill, Jessica Sewell, Samantha Lightner, Shreya Vodapalli, Yizhou Ma, Heather Bruce, Shuo Chen, Yunlong Tan, Peter Kochunov, L. Elliot Hong

**Affiliations:** 1 Maryland Psychiatric Research Center, Department of Psychiatry, University of Maryland School of Medicine, Baltimore, MD USA; 2 Psychology Department, University of Maryland, Baltimore County, Baltimore, MD USA; 3 Psychotic Disorders Division, McLean Hospital, Belmont, MA USA; 4 Department of Psychiatry, Harvard Medical School, Boston, MA USA; 5 Peking University HuiLongGuan Clinical Medical School, Beijing HuiLongGuan Hospital, Beijing, P. R. China

**Keywords:** Stress and resilience, Schizophrenia

## Abstract

Stress is implicated in psychosis etiology and exacerbation, but pathogenesis toward brain network alterations in schizophrenia remain unclear. White matter connects limbic and prefrontal regions responsible for stress response regulation, and white matter tissues are also vulnerable to glucocorticoid aberrancies. Using a novel psychological stressor task, we studied cortisol stress responses over time and white matter microstructural deficits in schizophrenia spectrum disorder (SSD). Cortisol was measured at baseline, 0-, 20-, and 40-min after distress induction by a psychological stressor task in 121 SSD patients and 117 healthy controls (HC). White matter microstructural integrity was measured by 64-direction diffusion tensor imaging. Fractional anisotropy (FA) in white matter tracts were related to cortisol responses and then compared to general patterns of white matter tract deficits in SSD identified by mega-analysis. Differences between 40-min post-stress and baseline, but not acute reactivity post-stress, was significantly elevated in SSD vs HC, time × diagnosis interaction *F*_*2.3,499.9*_ = 4.1, *p* = 0.013. All SSD white matter tracts were negatively associated with prolonged cortisol reactivity but all tracts were positively associated with prolonged cortisol reactivity in HC. Individual tracts most strongly associated with prolonged cortisol reactivity were also most impacted in schizophrenia in general as established by the largest schizophrenia white matter study (*r* = −0.56, *p* = 0.006). Challenged with psychological stress, SSD and HC mount similar cortisol responses, and impairments arise in the resolution timeframe. Prolonged cortisol elevations are associated with the white matter deficits in SSD, in a pattern previously associated with schizophrenia in general.

## Introduction

Schizophrenia spectrum disorder (SSD) is a severe mental illness characterized by psychosis, negative symptoms, and cognitive impairment. Its etiology remains unknown but likely includes stress-related pathophysiology, which is linked to the onset and exacerbation of psychosis, symptom severity [1], and prognosis [2]. Stress and adversity are also associated with elevated and dysregulated cortisol [3, 4]; and prolonged elevation of glucocorticoid levels is associated with brain functional and structural changes [5]. However, evidence bridging prolonged elevations of glucocorticoids, an abnormal stress response, and structural brain changes in SSD are limited.

White matter (WM) may be particularly vulnerable to pathogenic effects of stress, as oligodendrocytes and myelination are adversely affected by glucocorticoids [6]. Chronically elevated glucocorticoids impair WM reorganization following injury while promoting gliosis [7, 8]. Cortisol may also indirectly influence WM integrity through pro-inflammatory cytokines [9, 10] leading to neuronal and oligodendrocyte cellular and receptor level dysfunction and damaging myelination patterns in pre-clinical studies [8, 11]. In clinical studies, chronic stress is associated with WM lesions [12] and reduced fractional anisotropy (FA), an index of WM microstructural integrity using diffusion tensor imaging (DTI) [13]. Importantly, reduced FA is consistently associated with SSD patients [14], evident even in antipsychotic-naive first-episode patients [15] and in non-ill, first-degree relatives [16]. These WM changes are present in a regionally specific pattern, with the most affected WM tracts at the interhemispheric callosal and frontal fibers such as the corpus callosum (CC) and the anterior corona radiate (ACR), while other regions like the corticospinal tract (CST) are not [17, 18]. Although not traditionally thought of as tracts with direct stress responsiveness, these WM tracts connect prefrontal cortical and limbic structures that are responsible for mediating HPA-axis responses to stress [19, 20]. Tracts more specifically associated with limbic regional connectivity such as the fornix and the cingulate gyrus associated cingulum (CGC) are also among the more affected WM tracts in SSD [21, 22]. Abnormal cortisol reactivity in SSD could also represent a communication failure in these WM structures [23].

Despite extensive studies, the underlying mechanism of the decreased WM integrity in SSD remains elusive. We hypothesized that aberrant stress-induced glucocorticoid responses in SSD are partly responsible for these reported WM deficits. Furthermore, as discussed above, not all WM tracts are equally affected in SSD [18, 24]. If aberrant stress responses in SSD are contributory to WM deficits, we expect that regional tracts more vulnerable in SSD and/or more closely associated with limbic functions are also more strongly associated with the stress response abnormalities. Therefore, we hypothesized that inadequate or prolonged resolution of cortisol levels in response to stress would be associated with WM alterations in a tract-specific manner involving regions, in part, responsible for limbic communications previously mentioned.

We have previously examined the first hypothesis in 30 patients and 33 controls, although the sample size was limited and we did not find a statistically significant stress by diagnosis interaction [14]. With a larger cohort of participants, we re-examined the hypothesis and additionally examined the hypothesis that abnormal glucocorticoid response to psychological stress is associated with schizophrenia-related regional vulnerability patterns in the WM.

## Materials and methods

### Participants

Participants with SSD (*n* = 121) and HC (*n* = 117) completed the stress challenge task and DTI brain imaging; the sample included the 30 patients and 33 controls in the preliminary report [14]. SSD patients (*n* = 121: 84 males; age = 36.6 ± 13.3 years [SD]) were recruited from outpatient clinics at the Maryland Psychiatric Research Center and neighboring mental health outpatient clinics. HC (*n* = 117: 60 males; age = 35.4 ± 13.6 years) were recruited through media advertisements. Time interval between the psychological stress test and imaging session was 6.0 ± 8.0 days, with no significant group difference (*t* = −1.3, *p* = 0.2). The Structured Clinical Interview for *DSM*-IV or −5 was utilized to obtain diagnoses of either schizophrenia or schizoaffective disorder, categorized as SSD. Controls had no current DSM severe mental illness diagnoses, however past single episode depression was not exclusionary. Controls had no family history of psychosis in the prior two generations. Current or past major medical and neurological illnesses, history of head injury with cognitive sequelae, intellectual disability, substance dependence within the past 6 months, or current substance abuse were exclusionary for all participants. Substance use disorder was based on SCID interview, while urine toxicology screening was performed for verification purposes when indicated to confirm current substance use and allowed for excluding participants based on results. Clinical and cognitive assessments were performed using: Brief Psychiatric Rating Scale (BPRS) and Brief Negative Symptom Scale (BNSS) [25]; Digit Symbol Substitution test (for processing speed) from the Wechsler Adult Intelligence Scale-3 [26] and Digit Sequencing task (for working memory) from the Brief Assessment of Cognition in Schizophrenia [27]. Antipsychotic regimens for participants were converted into CPZ equivalents: 9 on both typical and atypical, 69 on atypical, 10 on typical, 19 on clozapine, one on clozapine and typical, seven on clozapine and atypical, and six not on any antipsychotic. Demographic and clinical characteristics are summarized in Table 1. Protocols were approved by the University of Maryland IRB and written informed consent to each participant in the study was collected.

**Table 1:** Participant demographic and clinical information.

	HC (*n* = 117)	SSD (*n* = 121)	Test statistic	*p* value
Age [years] (SD)	35.4 (13.6)	36.6 (13.3)	*t* = −0.73	0.47
Sex (% male)	52%	69%	*χ*^*2*^ = 7.78	<0.01
Current smoker (%)	32.5%	43.8%	*χ*^*2*^ = 3.23	0.048
BPRS^a^ (SD)	23.7 (4.2)	38.9 (10.7)	*t* = −14.20	3 × 10^−33^
BNSS^b^ (SD)	2.4 (5.7)	16.1 (14.4)	*t* = −9.48	4 × 10^−18^
Processing speed^c^	78.0 (16.5)	60.3 (19.9)	*t* = 7.29	5 × 10^−12^
Working memory^d^	20.5 (4.3)	17.4 (5.4)	*t* = 4.79	3 × 10^−6^
CPZ (SD)^e^	NA	495.5 (462.7)	NA	NA
Average Motion Distance per Frame (SD)	1.05 (0.53)	1.42 (1.32)	*t* = *−*2.72	0.007

^a^Brief Psychiatric Rating Scale (BPRS) calculation based on data from *n* = 116 SSD patients and 116 HC.

^b^Brief Negative Symptom Scale (BNSS) calculation based on data from *n* = 111 SSD patients and 116 HC.

^c^Digit Symbol Test for processing speed calculation based on data from *n* = 112 SSD patients and 115 HC.

^d^Digit Sequence Test for working memory calculation based on data from *n* = 113 SSD patients and 115 HC.

^e^Antipsychotic medication dose is provided in chlorpromazine equivalent dose (CPZ) (milligrams per day).

### Psychological stressor tasks

The psychological stressor tasks include the Paced Auditory Serial Addition Task (PASAT) [28] and the Mirror-Tracing Persistence Task [29], order randomly assigned and completed in series, to study an individual’s distress tolerability [30]. Procedures were followed as previously established [31]. Briefly, in the PASAT, a computer mouse was used to select the correct sum of consecutive numbers presented briefly on a computer screen. The MTPT asked participants to trace a dot along the outline of a star-shaped image on the computer using the mouse. Response errors were met with a loud, ~90 decibel, aversive buzzer noise. Learning sessions were performed prior to the experimental one. Speed and accuracy of responses were measured, and an algorithm automatically titrated the task presentation to provide similar challenges across participants. Participants were incentivized to continue, but could terminate the sessions at any point, coded in binary as: 1 = completed one or both tests, “distress tolerant”; 0 = quit both tests, “distress intolerant”.

**Fig. 1:**
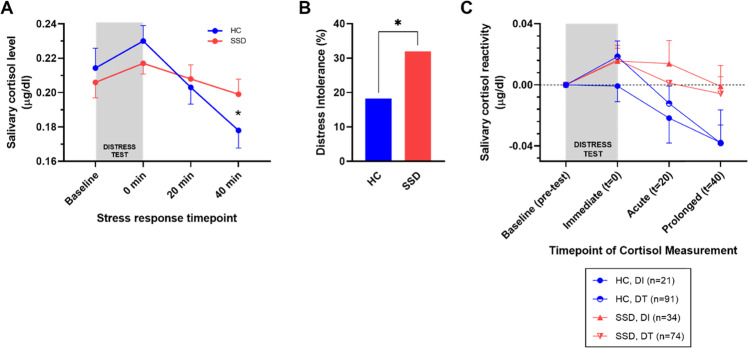
Psychological stressor task responses in cortisol levels and distress intolerance. **A** Salivary cortisol levels obtained at four timepoints, baseline, *t* = 0 min post-test, *t* = 20 min post-test, and *t* = 40 min post-test. Data represent mean (SEM) for each group. **B** Distress intolerance between the HC and SSD. **C** Salivary cortisol reactivity measured as subtraction from baseline with the respective timepoint. Cohorts were split into diagnosis and distress test outcome and reported as mean (SEM). HC healthy controls, SSD schizophrenia spectrum disorder, DT distress tolerant, DI distress intolerant; *p < 0.05.

Participants provided saliva at four timepoints: prior to task initiation (baseline), immediately (*t* = 0), 20-, and 40-min post-task completion/quitting (Fig. 1A). Thirty minutes before the baseline sample collection and after the post 0-min collection timepoint, participants sat comfortably, watched a scenic video, and/or read leisurely. All testing sessions were held approximately between 1200 and 1600 h. Participants refrained from eating, drinking, or smoking for 1-h before testing. Saliva samples were placed on ice and immediately stored after the test at −80 °C until assay. Prior to assay, samples were thawed and centrifuged at 10,000 *g* for 10-min. Cortisol was assayed using a commercial enzyme immunoassay kit (Salimetrics), following the manufacturer recommended protocol. Intra-assay coefficient of variance (CV) was 7.77% and inter-assay CV was 4.18% in our lab. Salivary cortisol levels (µg/dL) are highly correlated with serum levels and typically peak at 30 min following the initiation of a stressor task [32]. Physiological cortisol reactivities were calculated as the difference between the concentration obtained at baseline and 0-, 20-, and 40-min post-task completion, as the immediate, acute, and prolonged cortisol reactivity, respectively, in agreement with previously published studies [14, 33, 34].

### Diffusion tensor imaging (DTI)

All MR examinations were performed at the University of Maryland Center for Brain Imaging Research, using a Siemens 3T TRIO MRI (Erlangen, Germany) system equipped with a 32-channel phase array head coil. DTI data were collected using a single-shot, echo-planar, single refocusing spin-echo, T_2_-weighted sequence, with ‘GeneRalized Autocalibrating Partially Parallel Acquisitions’, acceleration factor 2 [35], yielding voxel dimensions 1.7 × 1.7 × 3.0 mm, acquisition time ~8 min. The sequence parameters were: echo time/repetition time = 87/8000 ms, field-of-view = 200 mm, axial slice orientation with 50 slices and no gaps, five *b* = 0 images and 64 isotropically distributed diffusion-weighted directions with *b* = 700 s/mm^2^. ENIGMA-DTI pipeline (https://www.nitrc.org/projects/enigma_dti) was used for tract-based spatial statistical analysis of diffusion anisotropy [36] with protocol demonstrating excellent reproducibility [37, 38]. FA images were created using previously published protocols [14, 39] from the JHU atlas [40] and then nonlinearly aligned to a group-wise, minimal-deformation target (MDT) brain using the FLIRT method [36]. Briefly, the diffusion tensor was fit to the motion and eddy current diffusion data. RMSDIFF [41] was used to estimate the root mean square (RMS) movement distance between diffusion sensitized and *b* = 0 images. The RMS difference was calculated by comparing two 4 × 4 transformation matrixes: a transformation matrix from each frame to the first b0 image and an identity matrix which served as the no-movement reference. The advantage of the RMS distance includes both translation and rotation effects, providing an index of motion for the whole brain [42]. Motion statistics are displayed in Table 1 and included in all covariate analysis. All data passed quality assurance control of 2.5 mm accumulated motion during the scan.

The QA/QC parameters were derived empirically as an adequate threshold in a test-retest examination of subject for motion, as described previously by our group and others [37]. The group’s MDT brain was identified by warping all individual brain images in the group to each other. Group-average FA image was used to create a group-wise skeleton of 24 bilaterally-averaged WM tracts, thresholded at FA = 0.20 level to eliminate non-WM voxels, and FA values were projected onto the group-wise skeleton to account for residual misalignment among individual WM tracts.

### Statistical analysis

Group differences on demographics and clinical measures were examined with ANOVA or *χ*^2^. The primary measure of cortisol reactivity in response to the stressor challenge was analyzed by repeated measures (RM)-ANOVA, where the four timepoints were the repeated measures, and diagnosis was the between-subject factor. Post-hoc analyses for significant diagnosis x time interaction were then performed between individuals and baseline. Greenhouse–Geisser corrections were used to report RM-ANOVA. Correlation analyses between WM FA and psychological stressor task measures were corrected for motion, age, sex, and current cigarette smoking status, all features known to affect WM FA, and reported as partial *r-*values. Fisher’s *r-*to*-z* transformation was performed to compare group difference in correlation coefficients. All significance was two-tailed. False discovery rate (FDR) was used for multiple comparisons of 24 WM tracts with a *q-*value set at <0.05.

For WM regional vulnerability assessment, the ENIGMA study provided a meta-analysis of the case/control effects associated with schizophrenia in 23 major WM regions (Cohen’s *d*) [17] that represent the expected pattern of disease effects across different WM tracts as established by the largest DTI study in schizophrenia [18], and can be found in Supplementary Table 1. Correlational coefficients from the correlation analyses between each WM tract and psychological stressor task measures in the current study was then correlated with the ENIGMA tract effect sizes, which investigated whether tracts associated with the psychological stress are more likely tracts also affected by schizophrenia in general as determined by the ENIGMA study. The abbreviations of the WM tracts are also included in Supplementary Table 2.

## Results

### Behavioral and glucocorticoid response to the psychological stressor

SSD and HC participants were similar in age (*t* = −0.72; *p* = 0.47), but not sex (69% vs 52% male, respectively; *χ*^*2*^ = 7.78; *p* < 0.01) or current smoking status (43.8% vs 32.5%, respectively; *χ*^*2*^ = 3.23; *p* = 0.048) (Table 1).

SSD participants were significantly more likely to be distress intolerant than HC (32% compared to 18%, respectively; *χ*^*2*^ = 5.80; *p* = 0.016) (Fig. 1B), in agreement with our preliminary report [31]. At baseline, cortisol levels were not significantly different based on group (*F*_*1,221*_ = 0.37, *p* = 0.55). SSD and HC similarly increased cortisol immediately following psychological stressor, followed by declines from 0 to 40 min post-stress, with group differences in the final timepoint (Fig. 1A). RM-ANOVA demonstrated a significant time (*F*_*2.3,499.9*_ = 4.4, *p* = 0.005) and time x diagnosis (*F*_*2.3,499.9*_ = 4.1, *p* = 0.013) interaction, but no significant diagnosis main effect (*F*_*1,217*_ = 0.018, *p* = 0.9).

To identify the key timepoint for this interaction effect, we performed post-hoc analysis with a series of RM-ANOVA tests comparing baseline with each timepoint. Between baseline and immediately post-stress showed a significant time effect (*F*_*1,236*_ = 7.83, *p* = 0.006) without diagnosis (*F*_*1,236*_ = 0.61, *p* = 0.43) or time × diagnosis interactions (*F*_*1,236*_ = 0.024, *p* = 0.88). Between baseline and 20-min post-stress, there was no significant time effect (*F*_*1,231*_ = 0.48, *p* = 0.49), diagnosis (*F*_*1,231*_ = 0.03, *p* = 0.86), or time × diagnosis interaction (*F*_*1,231*_ = 1.58, *p* = 0.21). Between baseline and 40-min post-stress, however, there was a significant time (*F*_*1,223*_ = 10.43, *p* = 0.001) and time × diagnosis interaction (*F*_*1,223*_ = 4.30, *p* = 0.039), but no diagnosis main effect (*F*_*1,223*_ = 0.26, *p* = 0.61). We directly compared this cortisol reactivity interaction (post-stress 40-min minus baseline) by group differences, and SSD had significantly higher (less reduced) relative cortisol response (*F*_*1,221*_ = 4.7, *p* = 0.03) (Fig. 1A), with no change once including sex and smoking as covariates (*F*_*1,218*_ = 4.84, *p* = 0.03). However, 3 cortisol reactivity values were >3 standard deviation (SD) from the mean (*n* = 2 HC, *n* = 1 SSD, 2 slightly above 3 SD, one at 4 SD) and removing them did change the significant group differences (*t* = −1.4, *p* = 0.15) although still in the same trend. As no outliers were present in the individual cortisol timepoints and the cortisol reactivity outliers were ≤4 SD, these samples were retained for further analysis. Distress intolerance was also added as a covariate (distress intolerance vs. non-distress intolerance) because it was significantly different between groups (Fig. 1B), in agreement with our preliminary analysis [14], but was not significant (*F*_*1,218*_ = 0.03, *p* = 0.85), and the prolonged cortisol reactivity effect remained (*F*_*1,218*_ = 5.9, *p* = 0.02). Distress intolerance then subdivided groups (Fig. 1C) but no significant diagnosis by distress intolerance (DI vs. DT) effect (*p* = 0.7–0.9) or distress intolerance main effect (*p* = 0.33–0.52) emerged at any of the timepoints, suggesting cortisol levels primarily tracked with diagnosis. Subsequent analyses focused on prolonged cortisol reactivity.

As a complementary analysis, differences of cortisol levels from its peak to the 40-min timepoint (peak minus 40 min) were also compared between groups, and controls (0.089 ± 0.0054) showed significantly larger decline in cortisol level compared to SSD (0.059 ± 0.0054) (*t* = 2.6, *p* = 0.009), again suggesting a slower decline from the peak levels in SSD.

### White matter and the prolonged cortisol response

**Fig. 2:**
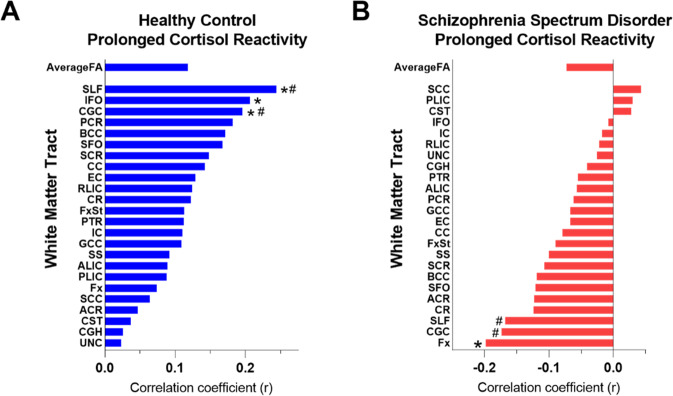
Correlations between prolonged cortisol reactivity and white matter tracks. **A** HC and **B** SSD. These tracts included the: genu, body, and splenium of corpus callosum (GCC, BCC, SCC, respectively), fornix (Fx), fornix-stria terminalis (FxST), internal capsule (IC), anterior and posterior limb and retrolenticular part of the internal capsule (ALIC, PLIC, and RLIC, respectively), external capsule (EC), corticospinal tract (CST), corona radiata (CR), anterior, posterior, and superior corona radiata (ACR, PCR, and SCR respectively), thalamic radiation (TR), posterior thalamic radiation (PTR), superior longitudinal fasciculus (SLF), inferior fronto-occipital fasciculus (IFO), superior fronto-occipital fasciculus (SFO), cingulate gyrus/hippocampus (CGH), cingulum cortex (CGC), uncinate fasciculus (UF), and sagittal striatum (SS). Asterisk indicates significant correlations between FA and cortisol reactivity in each group at *p* < 0.05; Hash indicates significant differences in the correlation coefficient between SSD and HC after FDR corrected significance with *q* < 0.05.

Prolonged cortisol reactivity in SSD was significantly correlated with whole-brain averaged WM FA (Suppl. Fig. 1A), (*r* = 0.22, *p* = 0.02). This significant association was reduced if excluding the three cortisol reactivity values that fell between 3 and 4 standard deviations of the mean (*r* = −0.1, *p* = 0.2) (Supplementary Fig. 1B). Tract-level correlational analyses was also performed, and several major WM tracts in controls (Fig. 2A) and SSD (Fig. 2B) were observed to be significantly correlated with cortisol reactivity. These include the SLF (*r* = 0.26, *p* = 0.006), IFO (*r* = 0.25, *p* = 0.01), and CGC (0.2, *p* = 0.04) in the HC group and the Fx (*r* = −0.23, *p* = 0.01) in the SSD group. In comparing group tract-level correlation coefficients, correlations most significantly differed in SLF (*z* = 3.03, *p* = 0.002) and CGC (*z* = 2.71, *p* = 0.006), as well as trend level significance (not surviving FDR correction) in BCC (*z* = 2.12, *p* = 0.03), superior fronto-occipital fasciculus (SFO) (*z* = 2.1, *p* = 0.03), and Fx (*z* = 2.0, *p* = 0.04).

We also used whole-brain FA as a covariate for further analysis (Supplementary Fig. 2). The general pattern was altered but HC still demonstrated positive correlations between the prolonged cortisol reactivity and WM FA in most of the tracts, while SSD demonstrated negative correlations in more of the tracts. However, no tract showed significant correlations after covarying out whole-brain average FA. Tracts were then rank ordered and compared to patterns generated with versus without including whole-brain average FA as a covariate. Their ranks were highly correlated in both HC (*r* = 0.85, *p* ≤ 0.001) and SSD (*r* = 0.92, *p* < 0.001). Lastly, a Fisher’s *r-*to-*z* transformation for the correlation coefficient differences was performed, revealing significant differences remained in 2 of the 6 tracts that were significant both with and without covarying whole-brain FA: CGC (*z* = 2.42, *p* = 0.01) and SLF (*z* = 2.8, *p* = 0.005) between HC and SSD. FA values are in Supplementary Table 1 along with effect sizes to compare with ENIGMA findings. A correlation analysis between the effect sizes of the tracts in our sample and those of the ENIGMA found that the tract-wise effect sizes of the current sample were significantly correlated to those reported in ENIGMA (*r* = 0.44, *p* = 0.03).

### Prolonged cortisol reactivity and white matter deficit patterns in schizophrenia

**Fig. 3:**
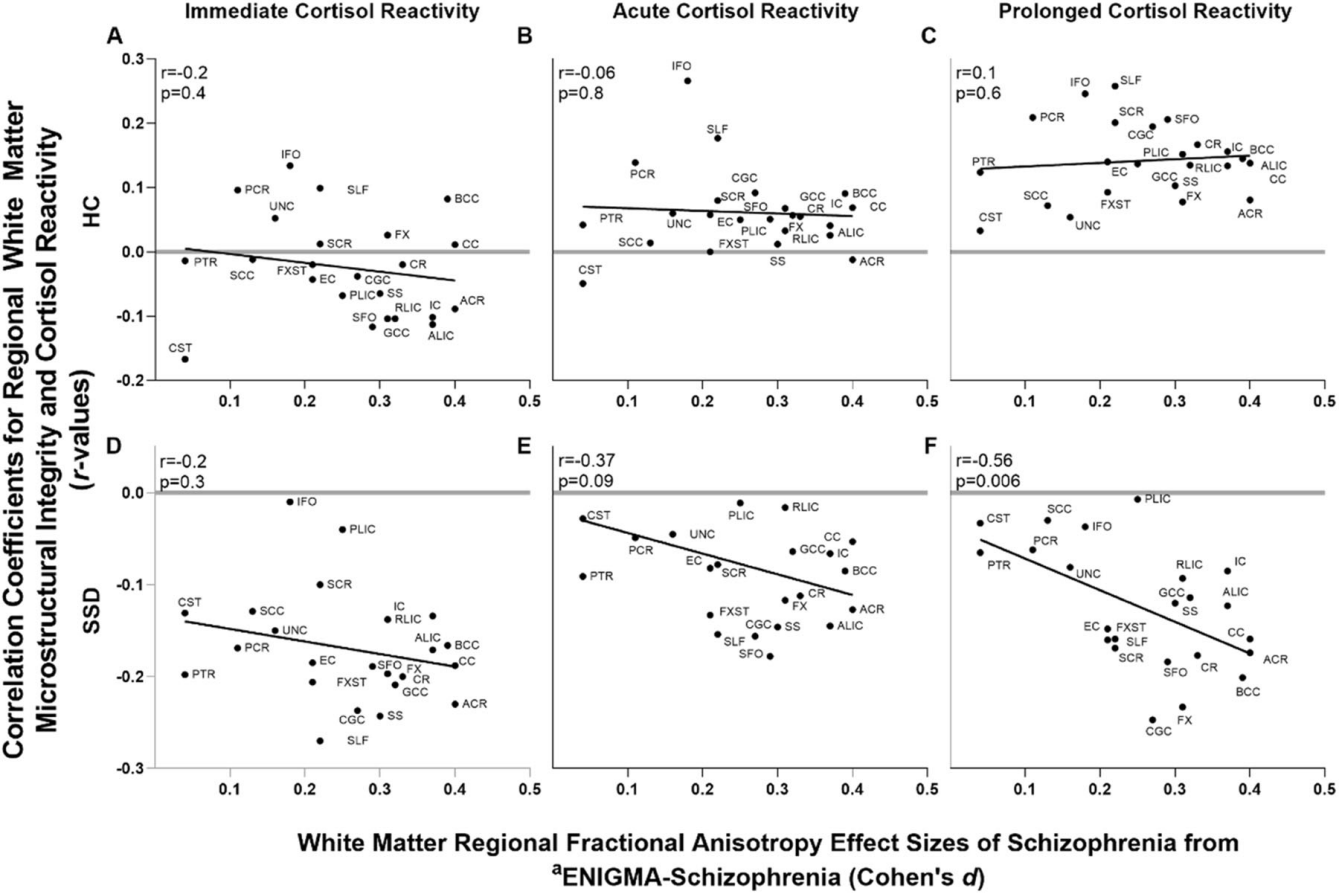
Prolonged cortisol reactivity effects on white matter tracts versus white matter tract findings in the ENIGMA study^a^. Response to stressor tasks measured with salivary cortisol at the immediate, acute, and prolonged reactivity timepoints in HC (**A**–**C**) and SSD (**D**–**F**). ^a^ENIGMA = Enhancing Neuro Imaging Genetics Through Meta-Analysis. Effect sizes of the 23 major white matter regions in the ENIGMA study are used, with higher values indicating more severe impairment in SSD patients compared with HC subjects in the ENIGMA meta-analysis (*x*-axis). Correlation coefficients between fractional anisotropy (FA) of each region and psychological stressor task measure in the present study are shown (*y*-axis).

As the ENIGMA-Schizophrenia DTI study provided the rank of WM tract effect sizes (Cohen’s *d*) affected by schizophrenia in general, we studied how they may be related to prolonged cortisol reactivity (Fig. 3). Correlations between prolonged cortisol reactivity and different WM tracts were significantly associated with ENIGMA tract effect sizes in SSD (*r* = −0.56, *p* = 0.006) (Fig. 3F). This appears specific to the prolonged cortisol reactivity, as immediate (Fig. 3D) and acute (Fig. 3E) cortisol reactivities did not show statistically significant associations. This relationship was unobserved in HC (Fig. 3A–C).

### Prolonged cortisol response and clinical characteristics

Distress intolerance was not significantly associated with whole-brain WM FA (*r* = 0.14, *p* = 0.16) or prolonged cortisol reactivity (*r* = 0.02, *p* = 0.86) in SSD. In SSD, prolonged cortisol reactivity was not associated with CPZ (*r* = 0.05, *p* = 0.64), age (*r* = 14, *p* = 0.16), sex (*r* = −0.02, *p* = 0.84), smoking status (*r* = 0.01, *p* = 0.92), BPRS (*r* = −0.03, *p* = 0.7), BNSS (*r* = −0.06, *p* = 0.5) or working memory (*r* = −0.06, *p* = 0.56). Prolonged cortisol, however, was significantly associated with processing speed (*r* = −0.23, *p* = 0.02), although this would not be significant after corrected for multiple comparisons. In HC, no significant correlations were found between prolonged cortisol response and age (*r* = 0.02, *p* = 0.9), sex (*r* = −0.05, *p* = −0.6), smoking (*r* = 0.02, *p* = 0.8), working memory (*r* = 0.04, *p* = 0.7), or processing speed (*r* = −0.04, *p* = 0.7). Furthermore, the correlation coefficients between cortisol reactivity and processing speed were significantly different between SSD and controls (*z* = −2.09, *p* = 0.036).

## Discussion

Our study found that the laboratory-administered psychological stressor task successfully induced cortisol secretion in SSD and HC similarly. At 40 min post-stress, SSD demonstrated a significantly elevated cortisol level compared to that of HC. This prolonged cortisol reactivity was significantly correlated with WM FA. Nearly all tracts were oppositely associated with prolonged cortisol reactivity between groups: positively associated in HC but negatively associated in SSD. These findings strongly mapped onto the schizophrenia-vulnerable tracts previously established by mega-analysis.

Results demonstrate that SSD patients have abnormally elevated cortisol responses in the prolonged timeframe following acute challenge, despite similar baseline and initial post-stress cortisol levels compared with HC. Cortisol levels in HC demonstrate the expected pattern of daytime diurnal decline [34] post-stress and drop rapidly after the peak, and reach well below the baseline level at 40 min, while in SSD the cortisol levels at 40 min post-stress essentially only return approximately to the baseline level (Fig. 1). Cortisol response to stress in SSD is controversial in the literature. Earlier diathesis-stress models [43] have employed the Trier Social Stress Test [44] to examine this hypothesis, although studies produced variable, inconclusive results [45–47]. Consistent with the diathesis-stress model, SSD demonstrates increased basal morning [48] and decreased overall daily cortisol levels [48], flattened awakening responses [49], and sensitivity to early life psychosocial stressors [50, 51]. Meta-analysis suggests SSD displays overall similar subjective responses to stress ratings and no group differences in cortisol response compared to healthy controls [52], consistent with our immediate or acute timepoint findings. Our results instead support a prolonged elevation of cortisol responses after psychological distress in support of the diathesis-stress model for SSD. Impairments in recovery and/or sustained cortisol release after psychological stressors may serve a key component of the diathesis-stress dysfunction in SSD, reconciling contradictory reports in the field.

Linking reduced FA and elevated prolonged cortisol responses in SSD here supports the association of chronic stress with WM lesions [12] and microstructural damage [13]. Regulating increased cortisol from stress demands integrates prefrontal, amygdalar, and hippocampal [19] regions, impaired in SSD [53]. Prolonged cortisol levels could represent failure of upstream HPA-axis structures to provide feedback inhibition [20]. Poor resolution of the initial cortisol response or prolonged release of additional cortisol after psychological stress, repeatedly occurring over time, may cumulatively lead to WM alterations in SSD shown here and across literature. Alternatively, WM deficits could be the original abnormalities impairing associative fibers such as the fornix, cingulum, frontal corona radiata, and the body of the CC connecting limbic, prefrontal, and the lateral hypothalamus [23, 54], leading to inadequately resolved cortisol responses to psychological stress. We found there to be significant group-level differences on comparing correlation coefficients between SSD and HC that were not present within the same group as significantly correlated WM tract and cortisol levels, for instance between the SFO or BCC. We interpret this finding in that there was still a relationship between WM and prolonged cortisol reactivity in these tracts, although the relationship was not strong enough to be statistically significant. However, the difference in this relationship was still significant between groups based on disease presence/absence.

Whole-brain average FA has been used as a covariate for tract-specific effects in some [17, 55] but not other [22, 56, 57] major DTI studies to account for a common variance in the microstructural integrity. Covarying whole-brain average FA markedly reduced and often eliminated significant group difference findings on specific tracts [17], as we have seen here, which are expected as whole-brain FA is an average of the values of multiple tracts. Meanwhile, we observed that the order representing the strength of tract-specific correlation coefficients between different regions and cortisol reactivity were largely retained with versus without the whole-brain FA covariate. The patient-control differences for their correlation coefficients remained significant in the CGC and SLF.

Understanding why only the prolonged, but not acute cortisol reactivity, was significantly correlated with FA may be clinically important. Pre-clinical models show that prolonged glucocorticoid exposure inhibits oligodendrocyte growth globally [6] and reduces myelin in a dose-dependent manner [7, 8]. Rats with maladaptive stress responses showed abnormally elevated cortisol levels and reduced FA following stress [55]. Meanwhile, acute cortisol elevations serve adaptive stress response mechanisms [56] crucial in neural plasticity [58] and activates key protein mediators for oligodendrocyte function [59]. Our findings between early and prolonged cortisol responses and WM appears consistent with these reports.

Opposite patterns of WM tracts and prolonged cortisol reactivity in SSD versus HC may reflect the mechanisms discussed above. Chronically elevated cortisol has been associated with widespread reductions in FA across WM tracts particularly in the CC and cingulate cingulum, in agreement with our findings presented here, and is observed in otherwise non-psychiatric patients recovered from hypercortisolemic states imparted from Cushing’s syndrome [5]. Oligodendrocyte damage is found ubiquitously throughout gray matter and WM regions following prolonged cortisol exposure [6]. Therefore, negative correlations across all tracts in SSD may suggest a reduced physiologic compensatory return of elevated cortisol levels back to baseline that, over time, impacts microstructure health globally. Indeed, in HC, this cortisol response is adequately regulated, and thus positive correlations between all WM tracts and the 40-min cortisol response may indicate physiologically adaptive cortisol effects on WM in response to stress.

WM deficits in SSD are regionally specific [17], although mechanisms of tract-level vulnerabilities remain unclear. Here, prolonged cortisol response was the only stressor-related measure associated with WM FA in SSD, also in a regionally specific pattern, and the two tract-specific patterns were significantly correlated (Fig. 3). These results suggest WM microstructural changes seen broadly in SSD may be related to abnormally prolonged cortisol responses from psychological stress. What is driving the relationship is the strong effects from tracts such as corona radiata, callosal, and fornix fibers that are critical connections between the limbic and the prefrontal functions [23, 54], but weak effects from motor tracts such as PLIC and CST. Similar deficit patterns were found in ENIGMA-schizophrenia samples [17, 53] and linked to core cognitive deficits, particularly processing speed [24]. Therefore, although the nominally significant correlation between processing speed and prolonged cortisol levels was not corrected for multiple comparisons, the fact that processing speed was the only measure among all the clinical measures we had explored that was nominally associated with the prolonged cortisol levels is intriguing. Findings from the current study provide new evidence that the regionally specific SSD FA patterns represented by the ENIGMA study results may in part be a consequence of unresolved stress effects on WM.

Our findings should be considered with several limitations. Antipsychotic medications may impact cortisol secretion; however, current dose was not significantly associated with our findings. Sleep has known effects on rhythmicity of cortisol secretion [60], which was not recorded and will be important to factor in future studies. We also noted that some of the tracts, such as the SFO, were significantly associated with the prolonged cortisol reactivity but did not belong to the list of those suspected to primarily mediate cortisol’s effects, and thus additional research is needed to the underlying mechanism of their associations. Past substance use disorder was unaccounted for in the current sample, which may impart dysfunction on the stress response and WM impairments. The experimental paradigm would benefit from more frequent timepoint gatherings to better understand the cortisol level changes with refinement. This study is cross-sectional, preventing causal interpretability. The positive correlation between psychological stress-induced cortisol levels and WM FA in healthy controls was not hypothesized. Although it is consistent with an adaptive mechanism, this idea requires further experimental testing. FA was the only index used for WM assessment as it is most sensitive to SSD deficits compared to other diffusion parameters [17]. The DTI acquisition protocol was made backward compatible with the legacy imaging protocol executed on the previous version of the Siemens 3T scanner, including weaker gradient and image reconstruction systems, with further details supplied in the methods section. Diurnal variability in neuroendocrine responses may exist despite many controlled aspects of the testing protocol. Roles as a patient vs control in clinical settings, motivational deficits, symptoms like paranoia, or the presence of a research staff may also act as confounders. In our distress task, we minimized staff roles by automating testing sequences.

In conclusion, prolonged cortisol elevations following psychological stressors may provide new perspectives to understand how stress response mechanisms are involved in SSD. Tract-specific SSD WM deficits are important stress-related factors. These observations may guide efforts identifying interventions where targeted management could extend a neuroprotective effect.

## Funding and disclosure

Support was received from NIH grants R01MH116948, R01MH112180, P50MH103222, and the University of Maryland/Sheppard Pratt Psychiatry Residency PSTP Program. LEH has received or is planning to receive research funding or consulting fees from Mitsubishi, Your Energy Systems LLC, Neuralstem, Taisho, Heptares, Pfizer, Luye Pharma, Sound Pharma, Takeda, and Regeneron. All other authors declare no financial interests that could represent a conflict of interest.

## Supplementary information

Supplemental Material
