# Supplementary material for: White matter in prolonged glucocorticoid response to psychological stress in schizophrenia
Source: Neuropsychopharmacology. 2021 Jul 1;46(13):2312–9. doi: 10.1038/s41386-021-01077-4 (PMC8580975; doi:10.1038/s41386-021-01077-4)
Supplement: Supplementary file 1 — Supplemental Material [file 41386_2021_1077_MOESM1_ESM.docx]

**Supplemental Figure 1. Prolonged cortisol reactivity and global tract-averaged fractional anisotropy (FA) in schizophrenia compared to healthy controls.** Scatter plot showing individual participants’ prolonged cortisol reactivity associated with whole brain averaged FA. Top panel (A) shows entire data set, while bottom panel (B) shows n=3 extreme values of prolonged cortisol timepoint removed.

**Supplemental Table 1. Fractional anisotropy values and group comparisons of the 24 separate white matter tracts and the whole-brain averages. Bold** = Statistically significant after FDR correction for 24 white matter tracts at q<0.05.

**Supplemental Table 2. Definitions of white matter tract abbreviations.**

**Supplemental Figure 2. Correlations between prolonged cortisol reactivity and white matter tracts controlling for whole brain average fractional anisotropy.** # = statistically significant tract-level correlation coefficient differences after Fisher *r-*to-*z* transformation, q<0.05.

**
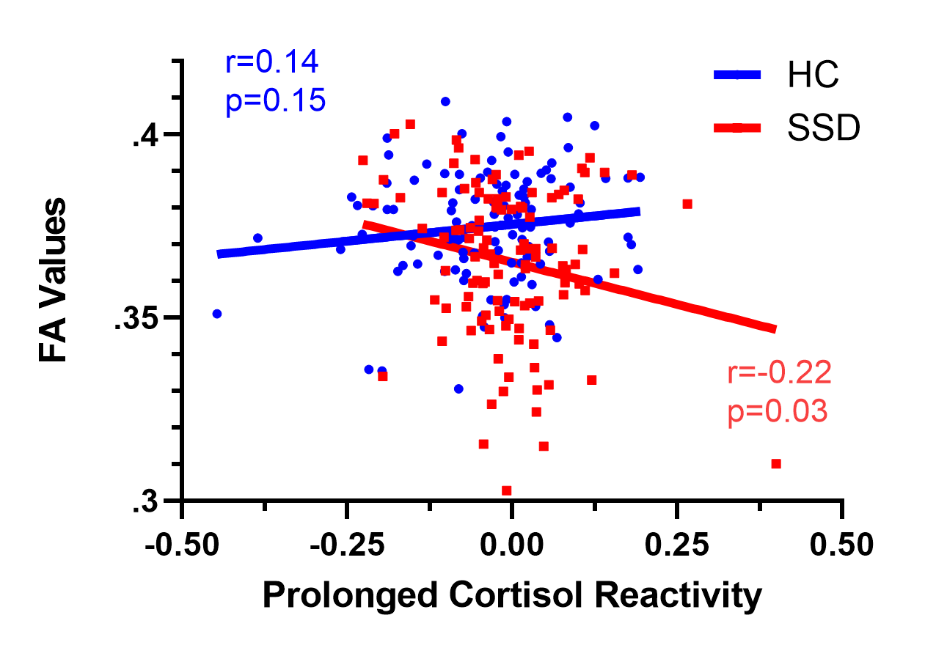

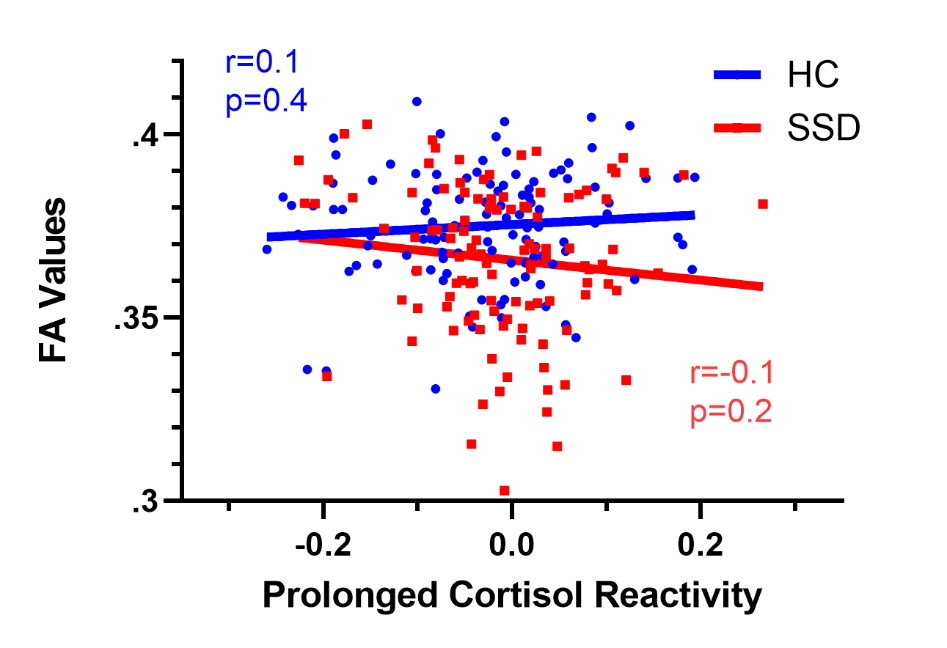
Supplemental Figure 1. Prolonged cortisol reactivity and global tract-averaged fractional anisotropy (FA) in schizophrenia compared to healthy controls.**

**B**

**A**

|  | **FA Values** | | | | | |  |  |
| --- | --- | --- | --- | --- | --- | --- | --- | --- |
| **White matter tract** | **HC (n=117)** | | **SSD (n=121)** | | **Test statistic (*F*)** | **p-value** | **Effect size (*d*)** | **ENIGMA Effect size (*d*)** |
|  | **Mean** | **SD** | **Mean** | **SD** |  |  |  |  |
| **Avg FA** | 0.3746 | 0.0153 | 0.3661 | 0.0210 | **12.98** | **.00039** | **0.47** | **0.42** |
| **CC** | 0.6985 | 0.0400 | 0.6745 | 0.0497 | **18** | **0.00032** | **0.53** | **0.40** |
| **BCC** | 0.6433 | 0.0477 | 0.6131 | 0.0660 | **17.74** | **3.6E-05** | **0.52** | **0.39** |
| **GCC** | 0.7290 | 0.0460 | 0.7046 | 0.0538 | **15.98** | **8.6E-05** | **0.49** | **0.32** |
| **ACR** | 0.4734 | 0.0324 | 0.4575 | 0.0374 | **14.97** | **0.00014** | **0.45** | **0.40** |
| **ALIC** | 0.5802 | 0.0324 | 0.5656 | 0.0351 | **13.42** | **0.00031** | **0.43** | **0.37** |
| **Fx** | 0.4582 | 0.0428 | 0.4365 | 0.0562 | **11.13** | **0.001** | **0.43** | **0.31** |
| **FxSt** | 0.5351 | 0.0325 | 0.5200 | 0.0376 | **9.99** | **0.002** | **0.43** | **0.21** |
| **SS** | 0.5515 | 0.0330 | 0.5368 | 0.0354 | **9.78** | **0.002** | **0.43** | **0.30** |
| **PTR** | 0.6040 | 0.0366 | 0.5886 | 0.0384 | **9.96** | **0.0018** | **0.41** | **0.04** |
| **CR** | 0.4856 | 0.0268 | 0.4745 | 0.0308 | **9.4** | **0.00024** | **0.38** | **0.33** |
| **EC** | 0.4857 | 0.0232 | 0.4766 | 0.0266 | **9.128** | **0.003** | **0.37** | **0.21** |
| **SCC** | 0.7497 | 0.0428 | 0.7347 | 0.0376 | **7.05** | **0.008** | **0.37** | **0.13** |
| **UNC** | 0.5525 | 0.0471 | 0.5369 | 0.0441 | **5.95** | **0.016** | **0.34** | **0.16** |
| **SFO** | 0.5210 | 0.0381 | 0.5084 | 0.0375 | **5.45** | **0.02** | **0.33** | **0.29** |
| **PCR** | 0.5022 | 0.0259 | 0.4934 | 0.0302 | **5.14** | **0.024** | **0.31** | **0.11** |
| **RLIC** | 0.5936 | 0.0328 | 0.5834 | 0.0337 | **6.36** | **0.012** | **0.31** | **0.31** |
| **CGC** | 0.6122 | 0.0406 | 0.5997 | 0.0441 | **7.21** | **0.008** | **0.3** | **0.27** |
| **IC** | 0.6163 | 0.0301 | 0.6079 | 0.0279 | **6.38** | **0.012** | **0.29** | **0.37** |
| **CGH** | 0.4906 | 0.0325 | 0.4803 | 0.0404 | **6.51** | **0.011** | **0.28** | **-** |
| **SLF** | 0.5034 | 0.0279 | 0.4956 | 0.0313 | **5.42** | **0.021** | **0.26** | **0.22** |
| **CST** | 0.5558 | 0.0303 | 0.5473 | 0.0384 | **5.397** | **0.021** | **0.25** | **0.04** |
| **SCR** | 0.4907 | 0.0272 | 0.4839 | 0.0327 | 2.32 | 0.13 | 0.23 | 0.22 |
| **PLIC** | 0.6663 | 0.0340 | 0.6664 | 0.0293 | 0.44 | 0.51 | 0.06 | 0.25 |
| **IFO** | 0.4934 | 0.0404 | 0.4921 | 0.0413 | 0.89 | .35 | 0.03 | 0.18 |

**Supplemental Table 1. Fractional anisotropy values and group comparisons of the 24 separate white matter tracts and the whole-brain averages.**

**Bold** = Statistically significant after FDR correction for 24 white matter tracts at q<0.05.

| **White Matter Tract** | |
| --- | --- |
| **Abbreviation** | **Definition** |
| **Avg FA** | Whole-brain average |
| **ACR** | Anterior corona radiata |
| **ALIC** | Anterior limb of internal capsule |
| **BCC** | Body of corpus callosum |
| **CC** | Corpus callosum |
| **CGC** | Cingulum |
| **CGH** | Cingulate gyrus |
| **CR** | Corona radiata |
| **CST** | Corticospinal tract |
| **EC** | External capsule |
| **Fx** | Fornix |
| **FxSt** | Fornix-stria terminalis |
| **GCC** | Genu of corpus callosum |
| **IC** | Internal capsule |
| **IFO** | Inferior fronto-occipital fasciculus |
| **PCR** | Posterior corona radiata |
| **PLIC** | Posterior limb of internal capsule |
| **PTR** | Posterior thalamic radiation |
| **RLIC** | Retrolenticular part of internal capsule |
| **SCC** | Splenium corpus callosum |
| **SCR** | Superior corona radiata |
| **SFO** | Superior fronto-occipital fasciculus |
| **SLF** | Superior longitudinal fasciculus |
| **SS** | Sagittal striatum |
| **UNC** | Uncinate fasciculus |

**Supplemental Table 2. Definition of white matter tract abbreviations.**


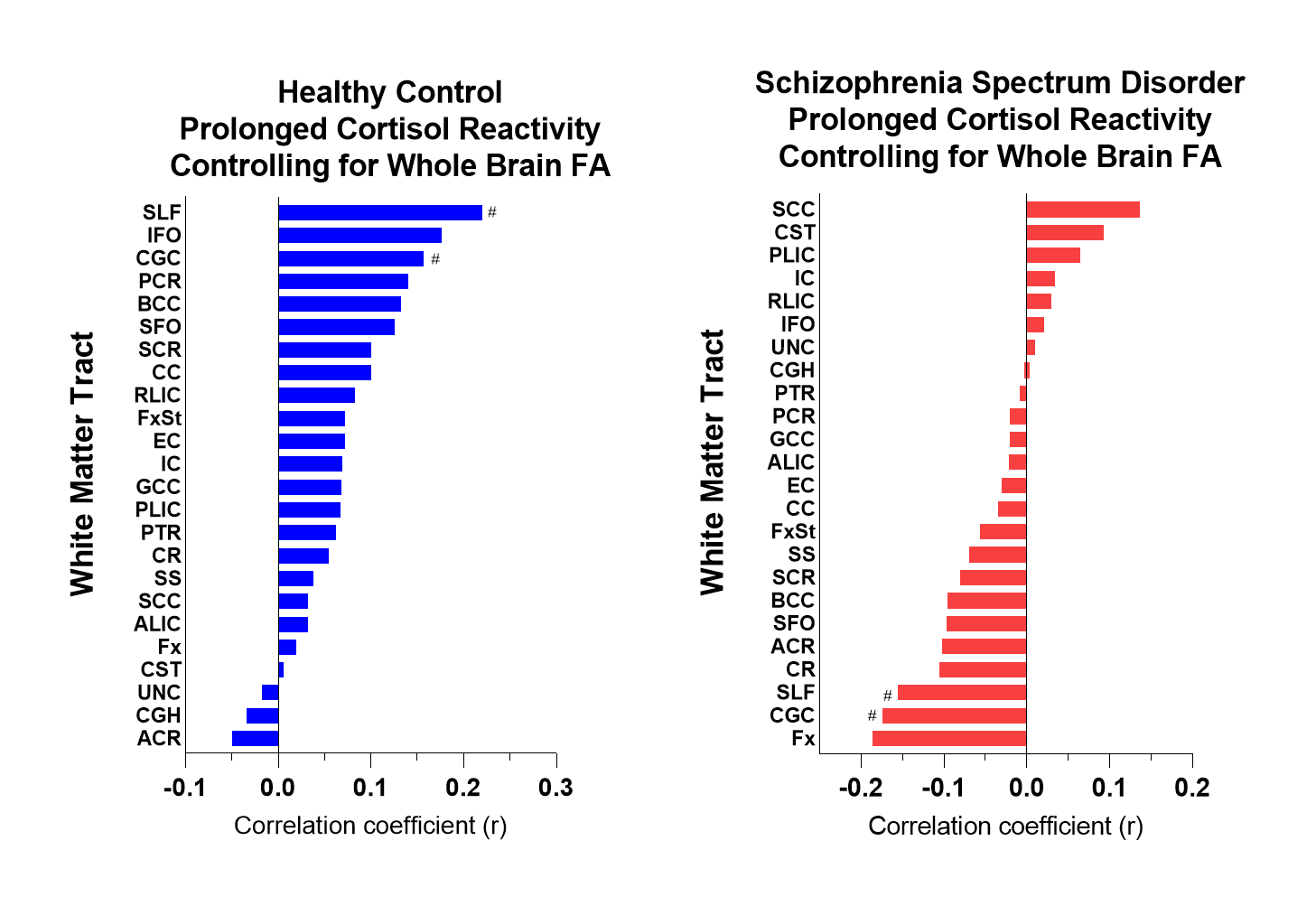
**Supplemental Figure 2. Correlations between prolonged cortisol reactivity and white matter tracts controlling for whole-brain average fractional anisotropy.**
